# Current State and Challenges of Tissue and Organ Cryopreservation in Biobanking

**DOI:** 10.3390/ijms252011124

**Published:** 2024-10-16

**Authors:** Irina V. Khaydukova, Valeria M. Ivannikova, Dmitry A. Zhidkov, Nikita V. Belikov, Maria A. Peshkova, Peter S. Timashev, Dmitry I. Tsiganov, Aleksandr V. Pushkarev

**Affiliations:** 1 Department of Refrigeration and Cryogenic Technology, Conditioning Systems, and Life Support Systems, Bauman Moscow State Technical University, 105005 Moscow, Russia; 2 Institute for Regenerative Medicine, Sechenov University, 119048 Moscow, Russia; 3 Russian Medical Academy of Continuous Professional Education, 125993 Moscow, Russia

**Keywords:** cryopreservation, biobanking, vitrification, biological tissues, organs, freeze–thaw

## Abstract

Recent years have witnessed significant advancements in the cryopreservation of various tissues and cells, yet several challenges persist. This review evaluates the current state of cryopreservation, focusing on contemporary methods, notable achievements, and ongoing difficulties. Techniques such as slow freezing and vitrification have enabled the successful preservation of diverse biological materials, including embryos and ovarian tissue, marking substantial progress in reproductive medicine and regenerative therapies. These achievements highlight improved post-thaw survival and functionality of cryopreserved samples. However, there are remaining challenges such as ice crystal formation, which can lead to cell damage, and the cryopreservation of larger, more complex tissues and organs. This review also explores the role of cryoprotectants and the importance of optimizing both cooling and warming rates to enhance preservation outcomes. Future research priorities include developing new cryoprotective agents, elucidating the mechanisms of cryoinjury, and refining protocols for preserving complex tissues and organs. This comprehensive overview underscores the transformative potential of cryopreservation in biomedicine, while emphasizing the necessity for ongoing innovation to address existing challenges.

## 1. Introduction

Cryopreservation is effective for long-term cell storage [1]; however, preserving tissues and organs is challenging due to deep-subzero injuries [2]. Static cold storage is the standard method for organ preservation, but limited storage time affects transplant success [3]. Approaches like normothermic and hypothermic machine perfusion extend organ viability and have been used in the transplantation of kidneys [4]. Supercooling and subnormothermic machine perfusion have also extended ex vivo organ viability by 27 h [5]. However, effective methods for preserving organs beyond 3–12 h are not yet in clinical practice [6]. Increased preservation time could expand transplant possibilities and facilitate pre-transplant preparation [6,7]. Further research is needed to address tissue and organ biobanking challenges [2].

Improving cryopreservation can aid regenerative medicine by preserving cells that are currently used immediately or after brief refrigeration [8]. Brain and lung tissue biobanking would ensure safety and support translational research [6,9]. Cryopreservation is vital for fertility preservation, allowing long-term storage of gametes and embryos, thus advancing reproductive medicine [6,10]. Cryopreservation is also pivotal in biodiversity preservation [11,12] and enhances hypothermic emergency medicine in resource-limited settings. A better understanding of cryopreservation effects can improve cryogenic protocols [6]. While cell cryopreservation is well reviewed, the literature on tissue and organ preservation often focuses on specific tissues [13,14]. This review aims to provide a comprehensive overview of techniques, success rates, challenges, and potential solutions.

## 2. Current State of Tissue and Organ Cryopreservation

While some samples, such as biological fluids [15], reproductive cells [16,17], and mesenchymal stromal cells [18], are relatively straightforward to cryopreserve using established protocols, other samples may require more refined techniques.

**Figure 1:**
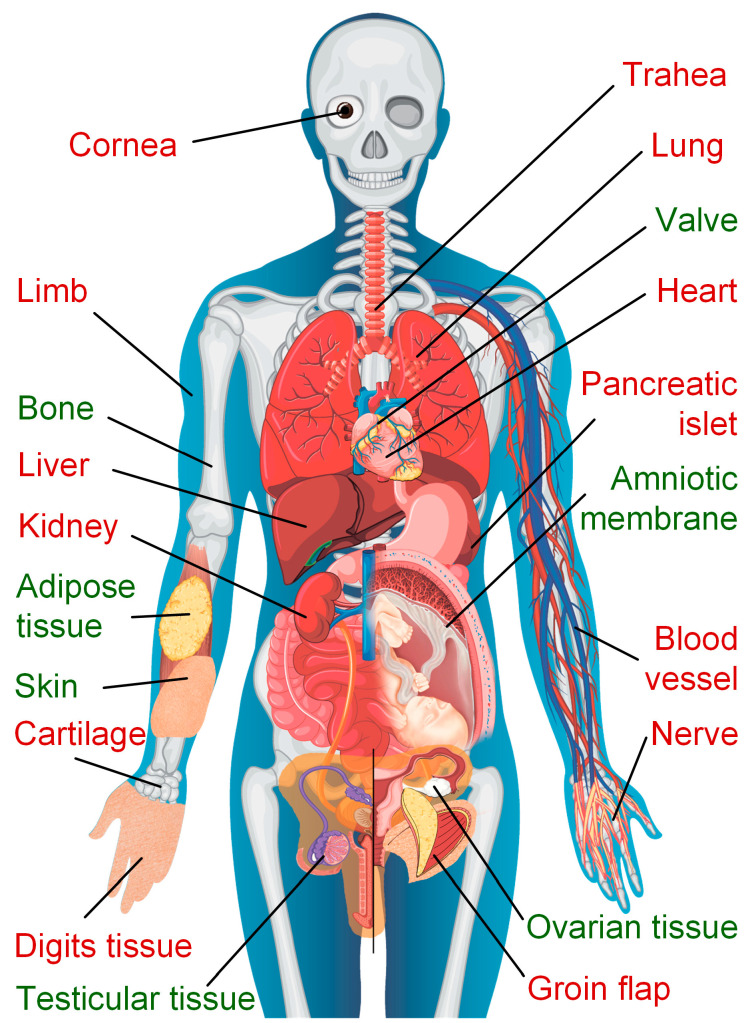
The tissues and organs that have been researched for successful cryopreservation [20]. The green captions denote the tissues that are routinely used in clinical practice and available in biobanks. The red ones represent tissues and organs that are currently only under research but have shown successful results.

Kidneys and hearts have been the most widely studied organs, but neither have been consistently recovered after cooling to temperatures lower than −45 °C [6,19]. Figure 1 shows the tissues and organs that have been researched for successful cryo-preservation.

### 2.1. Ovarian Tissue

Cancer treatment often causes gonad toxicity, leading to male and female infertility [6]. In women, cryopreservation preserves embryos, oocytes, or ovarian tissue. Ovarian tissue cryopreservation (OTC) and transplantation preserve many follicles with less cryoinjury than mature oocytes and require no ovarian stimulation [21]. This is crucial for prepubertal girls and women with hormone-dependent cancers who cannot delay treatment [10]. OTC is also being explored as a method to delay menopause [22].

The process involves harvesting and freezing the ovarian cortex or tissue strips. After thawing, the tissue can be transplanted back, or oocytes can be matured in vitro.

The effectiveness of the method is mainly assessed by oocyte viability, clinical pregnancy rate, live birth rate, and miscarriage rate.

Currently, no standardized OTC processing method exists, and a standardized human ovarian tissue vitrification protocol is still needed [10]. Slow freezing and vitrification are used. Most clinical studies have been based on slow freezing, examining live birth, pregnancy, and endocrine restoration. Vitrification has been used to a lesser extent for ovarian tissue. Slow freezing involves controlled cooling and immersion in liquid nitrogen, while vitrification requires quick equilibration and submersion [23,24,25]. Slow freezing uses different cryoprotectants and protocols. As an example of a generalized effective protocol that gives about 85% intact oocytes, the following can be given: mixing of cryoprotectant components for about 90 min, freezing to an intermediate temperature of −7 °C for 12 min, holding with further seeding and freezing to −150 °C, storage at −90 °C, thawing at room temperature for 30 min with a subsequent increase in temperature to 37 °C.

Depending on the cryoexposure plan, more or less damage to ovarian and vascular tissue or death of primordial follicles may be induced, so the selection of the best cryoexposure procedure is also the key point of clinical research work [26]. Studies show that combining CPAs with antifreeze polymers reduces toxicity in animal ovaries, but human data are limited [10]. Vitrification uses combinations of EG, DMSO, PrOH, and GLY, along with non-permeable CPAs like sucrose or trehalose [10]. Lowering CPA concentrations with DMSO improves follicular recovery. Another approach is the liquidus tracking technique, which gradually changes temperature and CPA concentration.

Success with small tissue samples aids rapid revascularization post-grafting but limits extrapolation to larger tissues. The largest samples are 8–10 mm × 5 mm strips [27], enabling techniques like ultra-rapid freezing [28] and needle-immersed vitrification [29]. However, freezing damage and ischemic issues hinder whole-ovary preservation [25,30].

Directional freezing can be considered as a promising method. It shows promise, with better results in sheep whole-ovary freezing compared to slow freezing [30].

### 2.2. Testicular Tissue

Cryopreservation of immature testicular tissue (ITT) is recommended for prepubertal boys undergoing gonadotoxic treatments, such as chemotherapy, as a fertility preservation method [6,31]. It maintains higher spermatogonial stem cell (SSC) viability compared to freezing testicular cell suspensions [13]. Many biobanks offer ITT cryopreservation [32,33], but it remains experimental due to the lack of reported human live births [34], despite successful animal studies [35].

Research on ITT cryopreservation focuses on developing effective human fertility preservation protocols and biodiversity preservation for various species. Slow freezing with seeding is the most common method [13].

Both controlled and uncontrolled slow freezing are used. If we consider human tissue, then the following can be given as an example of a generalized effective protocol for controlled freezing: the mixing of cryoprotectant components in an ice environment for 30 min, cooling to 0 °C and holding for 5 h, followed by a temperature decrease to −8 °C for 16 min, seeding, temperature decrease to −40 °C for 64 min, holding for 10 h, decreasing to −70 °C for 5 h and placing the object in liquid nitrogen for storage; after storage, thawing at 37 °C for 2 h, followed by washing for 5 h twice. This protocol effectively preserved the architecture, the number of tubules, SSCs, and Sertoli cells of prepubertal testicular tissues.

The small sample size (~3 mm^3^) allows for various protocols, including solid surface vitrification, open-pulled straws, and single-drop vitrification [35,36]. DMSO and sucrose are the typical permeating and non-permeating CPAs used.

A promising approach could involve developing new protocols for controlled slow freezing and vitrification using less toxic cryoprotectants to reduce washing time and improve the quality of the resulting material.

### 2.3. Heart

Heart transplantation is the most effective surgical treatment for end-stage heart failure. Static cold storage is the standard organ preservation technique, while newer methods like machine perfusion benefit high-risk cases and donation after circulatory death [3]. Cryopreservation of the heart is challenging due to the risk of cryoinjury, with 7% of autopsies on patients with primary graft dysfunction showing such injury [37]. Cryopreservation of small animal hearts frozen to −30 °C has shown that younger organs could sometimes be reanimated [38]. Human heart vitrification and nanowarming simulations require individualized thermal protocols to prevent damage [39]. While separate tissues, such as the pericardium [40] and myocardium [41], have been cryopreserved, the effects on pericardium properties have not been studied, and no similar articles were found.

There is currently no effective protocol for long-term cryopreservation of the heart.

As a promising method, it is possible to consider the creation of new protocols based on perfusion and controlled freezing. At the same time, it is more likely that the need to adapt the cryopreservation protocol for each specific case will remain due to the difficulty of determining the parameters of mechanical stress in advance inside the heart.

### 2.4. Heart Valve

Heart valves are primarily used in pediatric cardiac surgery to treat congenital malformations such as tetralogy of Fallot, valve atresia, bicuspid aortic valve, and complex destructive root anatomies [42,43,44,45]. The clinical use of heart-valve allografts is favored for their anatomical fit, reducing the need for external materials and minimizing infection rates [46]. Their similar hemodynamic properties ensure undisturbed blood flow [44,45] and no side effects of anticoagulation therapy due to their non-thrombogenicity [47].

Cryopreserved heart valves are a standard biobanking procedure, enabling long-term storage and analysis. Studies in the USA [48] and Brussels [43] reviewed the long-term risks and complications of allograft infections over 20 and 30 years, respectively, while other authors raised questions about heart valve storage duration. In Croatia, cryopreserved heart valves showed 53% and 47% 1-year and 3-year survival rates in infective endocarditis patients, with no graft reinfections [48].

A controlled slow freezing protocol was used, consisting of the following stages: the mixing of cryoprotectant components at 4 °C for 30 min, freezing to −40 °C for 44 min, then to −100 °C for 12 min, moving to liquid nitrogen and storing for 5 years, thawing at room temperature for 5 min with a transition to +37−40 °C. The practical implementation of the protocol indicates the possibility of long-term storage of heart valves, but the results of 1- and 3-year survival show the need to improve cryopreservation methods and protocols.

The Czech National Allograft Heart Valves Bank extended the expiration limit of cryopreserved valves based on mechanical and structural properties [49], using histological and mechanical testing to validate valve integrity [44].

As a promising method, it is possible to consider the creation of new protocols based on the use of minimally toxic or non-toxic cryoprotectants with the possibility of using controlled freezing.

### 2.5. Vascular Tissue

Vascular allografts from deceased donors are preferred for their compliance and histocompatibility, with donation and transplantation activities continuously increasing [50,51]. Cryopreserved allografts are used in hepatic vein [52], aorto-bi-iliac, and aorto-bi-femoral reconstructions, hepatobiliary surgeries [52], aortic valve endocarditis [53,54], kidney transplantation [55], and abdominal aortic infections [56]. Improper cryopreservation can cause allograft rupture due to microfractures during fast thawing [57]. Standardized cryogenic storage methods are needed to meet clinical demands effectively [57,58,59].

US and European storage protocols [60] have improved clinical outcomes. Studies using tissues cryopreserved in different CPAs have evaluated RNA integrity [61], and cryopreserved microvascular fragments have shown effective in vivo vascularization without differences in network formation [62]. However, cryopreserved allografts can deteriorate [63], with certain protocols causing early ruptures and life-threatening complications. Equilibrium and slow freezing protocols reduce these risks [52].

In these protocols, DMSO or a mixture of different components with DMSO is mainly used as a cryoprotectant. Options include one-, two-, or three-stage sequential freezing followed by storage in liquid or vaporous nitrogen for 1–5 years, thawing at 0 °C or room temperature with a transition to 37–40 °C.

Large studies reported 30-day mortality rates of 8.9% for aortic valve replacement [64] and 14.9% for aortoiliac infection [65], with 5-year survival rates of 77.4% for aortic-valve replacement [64] and 66.5% for homograft aortic root replacement.

As a promising method, it is possible to consider the creation of new protocols for controlled semi-automatic or fully automatic freezing based on the use of minimally toxic or non-toxic cryoprotectants [66].

### 2.6. Pancreatic Islet

Pancreatic islet transplantation treats diabetes but often fails to achieve insulin independence [67,68,69,70,71] and is limited by islet shortages and increased immunogenicity [70]. Cryogenic banking of islets from multiple donors can enhance availability and reduce immunogenicity [68,69]. Slow freezing allows the processing of large quantities of islets, but vitrification is preferred for small tissues to prevent cryoinjury and impaired insulin secretion [67,70]. Stepwise CPA addition during freezing mitigates its detrimental effects on cells [67,69].

In these protocols, DMSO or a mixture of different components with DMSO is mainly used as a cryoprotectant. After freezing, storage is mainly carried out in liquid or vapor nitrogen.

Despite advances, cryopreserved islet recovery remains low due to damage during thawing and rewarming, making these islets clinically inapplicable.

As a promising method, it is possible to use minimally toxic or non-toxic cryoprotectants in protocols or add oxygenation during these processes to reduce islet damage [68].

### 2.7. Adipose Tissue

Fat or lipoaspirate cryopreservation is used in fat grafting for facial and hand rejuvenation, augmentation, scar correction, and chest irregularities [72,73]. Storing larger amounts of harvested fat reduces the number of procedures needed. Adipose tissue and derived products have well-established biobanking procedures [72,74], and the success of adipose aspirate cryopreservation has been demonstrated in many patients [72,73]. However, an optimal protocol for plastic and reconstructive surgery is still needed.

The most common storage method is at −196 °C in liquid nitrogen (LN) [72,73,75]. While DMSO was originally used, a combination of DMSO and trehalose has been found effective, reducing the required amount of DMSO [76]. Trehalose alone may ensure cryoprotection without the need for DMSO washing [77].

Methods of controlled slow and uncontrolled freezing are used. Controlled slow freezing consists of mixing cryoprotectant components for 10–15 min at a temperature above 0 °C, freezing to −30–80 °C at a rate of 1–2 °C/min, followed by holding for 10 min or immediately placing in liquid nitrogen for storage. After storage, there is a one- or two-stage thawing with a final temperature of 37 C. Uncontrolled freezing is mainly carried out to −18–20 °C, followed by warming up to 37 °C for 6–10 min.

Adipose tissue can also be processed into stromal vascular fraction (SVF) gel, which is rich in stem cells and extracellular matrix [78]. The successful injection of cryopreserved SVF gel has been demonstrated in 425 patients with minimal complications.

As a promising method, it is possible to use minimally toxic or non-toxic cryoprotectants in controlled freezing protocols.

### 2.8. Amniotic Membrane

Human amniotic membrane (HAM) grafts are widely studied as scaffolding materials for skin, bone, cartilage, vascular, ureteral, vaginal, and periodontal regeneration [79,80,81], as well as for foot, ankle [82,83], and ocular surface diseases [84]. While most cryopreservation techniques use pulverized HAM for scaffold fabrication via allogeneic grafts, hydrogels, bioprinting, or electrospinning [80], whole tissue preservation is also being studied [85,86]. The common protocol involves freezing and storing HAM at −80 °C in glycerol, which can cause cell damage due to ice crystal formation.

Protocol optimization is needed based on the application. For instance, a study found that for ocular reconstruction, storage at −80 °C is as effective as using DMSO in LN [87]. Additionally, optimizing the thawing process is crucial to prevent the loss of soluble proteins important for wound healing [88].

As a promising method, it is possible to use minimally toxic or non-toxic cryoprotectants to avoid the potential toxicity of the DMSO-based freezing environment. Possible media include a dextran-based medium at −80 °C, dry freezing at −80 °C (no medium), or polydimethylsiloxane [89].

### 2.9. Cornea

The corneal endothelium, crucial for transparency [90], has limited regenerative capacity and is highly susceptible to cryoinjury, resulting in poor integrity and viability compared to fresh corneas [91]. Even though some centers report positive outcomes [92], eye banks do not routinely use corneal cryopreservation [6]; they store donor tissue hypothermally or by organ culture [93]. However, there are millions of patients worldwide who are waitlisted for corneal transplantation, which could benefit a significant majority of patients with visual impairment [92]. Therefore, research on corneal cryopreservation is ongoing. For example, cryopreserved corneas have shown benefits for treating ulcers in elderly or unwell patients [94].

While whole cornea cryopreservation remains challenging, protocols for preserving lenticules after the SMILE procedure show promise for various conditions due to low immunogenicity and simpler storage [95]. A review on lenticule cryopreservation up to 2020 revealed high success in their cryopreservation, including restoration of transparency and intact collagen structure using DMSO and FBS [96]. Less favorable results regarding neural density [97], impacted nerve regeneration after implantation, collagen fibrils, and interfibrillar distance [98] were reported with other CPAs.

The investigation of mechanical properties of corneal tissue could potentially advance the understanding of degeneration during cryoinjury, but testing small, fragile eye tissue is complicated [87].

### 2.10. Kidney

Kidney biobanking is crucial for addressing organ shortages. Techniques like hypothermic machine perfusion, normothermic oxygenated perfusion, and supercooling extend transplantation time. Among cryopreservation methods, slow cooling causes convective failures, while rapid LN plunging leads to cracks [99,100]. Joule heating has also been proposed for kidneys, but it requires separation into thin kidney slices and high CPA concentrations for effective heat diffusion [101]. Therefore, vitrification is the most promising method [19,99,100,102,103,104].

Vitrifying whole organs is challenging due to their size, requiring uniform and rapid rewarming to prevent ice crystallization. For very small kidneys, however, successful vitrification is possible. Cryopreservation of embryonic kidneys (metanephroi) has yielded successful results in regenerative medicine [103,104,105,106]. Vitrified kidneys were successfully transplanted into mice [104].

For larger organs, nanowarming, using radiofrequency-heated silica-coated iron oxide nanoparticles (sIONPs), is showing promise [99], preserving kidney histology. Good results have been demonstrated in a rat model with VS55 as a CPA [107]. Studies found that nanowarming perfusion at room temperature is more effective than at 4 °C [100], though higher temperatures can increase CPA toxicity. Nanowarming can also damage delicate kidney capillaries, impairing function [100].

### 2.11. Liver

Despite liver transplantation being the best option for many liver diseases [89,108], the supply of organs falls short of demand [109]. Hypothermic storage limits viability to 12–24 h, restricting the time available for screening and matching. Supercooling prevents ice damage at subzero temperatures but struggles with larger organs, which are prone to ice nucleation damage [110]. Modified supercooling has preserved rat livers at −6 °C [111] and human livers at −4 °C with protective agents [5,110], extending viability by 27 h [5].

Slow freezing causes ice crystal formation, making it ineffective for liver preservation due to the organ’s complexity. Vitrification is promising but faces challenges related to heat and mass transfer, toxicity, and rewarming [109,112]. Long-term storage and auxiliary transplantation of a pig liver using directional freezing have been reported [93]. Nanowarming with radio frequency magnetic fields and magnetic nanoparticles improves warming rates and uniformity of warming [113], successfully vitrifying and rewarming the largest organ to date—a rat liver—using ethylene glycol and sucrose [109]. However, scaling this method to human organs is limited by batch size and cost [108].

### 2.12. Cartilage

Articular cartilage (AC) injuries, lacking blood vessels, lymphatic vessels, and nerves, struggle to self-repair, often leading to osteoarthritis and joint disability [105,114,115,116]. Current hypothermic storage methods can preserve osteochondral allografts for only up to 28 days [117]. Cryopreservation addresses the limitations of short storage times and suboptimal tissue quality [115]. The main CPA used is DMSO both in slow freezing and vitrification, which yields good results. In vitrification, the CPA cocktail is more complex and gradual, usually including PrOH. Comparisons between fresh, frozen, and vitrified AC in a porcine model revealed no significant differences in mechanical parameters during rapid loading [115], consistent with other studies [114,118]. Nanowarming of vitrified samples shows promise over conventional warming methods, preserving more live cells and maintaining higher metabolic activity, mechanical properties, and structural integrity [116].

### 2.13. Bone

Cryopreserved autologous bone can be used in cranioplasty for its biocompatibility. The method for cryopreservation in cranioplasty typically does not involve any CPA. However, preserved autologous cranioplasties result in up to a 20% rate of reoperation [119] and can cause donor site morbidity [120]. Cryopreserved bone allografts used to replace large segmental bone defects remain largely avascular, resulting in a relatively high incidence of complications [121,122]. The rate of complications can be lowered using surgical angiogenesis [121], although some researchers suggest that this may be coupled with additional therapies [123]. Cryopreserved bone with viable cells can serve as a filler for mandibular augmentation [124].

There is also interest in the cryopreservation of bone marrow containing large quantities of hematopoietic stem and progenitor cells and mesenchymal stem cells [125]. The problem in that case is the impregnation of CPA inside the bone.

Dental experts explore the cryopreservation of teeth for the future of stem cell therapy. However, limitations such as low viable stem cell counts post-thawing and deformation of cytoplasmic shape hinder its clinical use [126]. Several approaches that can improve teeth cryopreservation are the use of magnetic fields and antifreeze proteins. Cooling in a magnetic field and DMSO improved cell survival and proliferation [127]. Vitrification with low-concentration antifreeze proteins has been also proposed for tooth cryopreservation as a hypothesis [128].

### 2.14. Trachea

To date, the surgical management of patients suffering from severe tracheal lesions that are not eligible for tracheal resection still represents a significant challenge, and research continues towards the identification of an effective tracheal circumferential substitute [129]. Tracheal allotransplantation has complications such as stenosis or graft necrosis and was gradually discarded [130]. Successful tracheal autotransplantation correlates with graft length [131,132], with shorter allografts showing better in vivo results. The use of a cryopreserved allogeneic aorta was also documented to be a viable source of tracheal replacement [133]. However, there are too few results to confirm that. Therefore, three alternatives with reduced immunogenicity and thus suitability for cancer patients present a possible option for prostheses: cryopreserved tracheal allografts, decellularized tracheal substitutes, and a combination of two technologies—epithelium-denuded-cryopreserved tracheal allografts [89,129,131,133]. All of the cryopreserved choices showed satisfactory results in animal studies. In a rabbit model, DMSO as a CPA resulted in good stiffness and vascularization, with 21% of animals dying from complications [131]. A pig model with DMSO/HSA demonstrated no cytotoxicity and similar compressive behavior to a control [129]. Neo-trachea fabrication using pre-epithelialized cryopreserved allografts has also been explored for viability and rigidity [132]. Unfortunately, for extensive resections, both biological and prosthetic materials have not yielded favorable outcomes [128].

### 2.15. Complex

There is relatively little successful research on complex tissue, containing several different tissues or organs. Composite tissue banking via cryopreservation offers an alternative to various perfusion methods such as static cold storage, usually, hypothermic machine perfusion, normothermic machine perfusion, ex vivo lung perfusion, the Organ Care System, and the two-layer method for kidney, liver, lung, heart, and pancreas cryopreservation [134].

Various solutions such as Collins solution, Euro-Collins solution, UW solution, HTK solution, Celsior solution, ET-Kyoto solution, polysol solution, Shanghai multiorgan solution, DMSO, sucrose, and polyvinylpyrrolidone have been used for vascularized composite tissue cryopreservation [135], with only DMSO with trehalose or sucrose proven effective in clinical practice [136,137].

Cooling rates vary depending on cell type. Because of the heat capacity, it is extremely difficult for larger objects to achieve ultrafast cooling rates [135]. However, vitrification is possible in combination with directional freezing [136,137]. Successful results have been demonstrated on rat hind limbs.

The right combination of CPAs even allows successful cryopreservation of such a composite organ as a digit [138]. Finger transplantation is effective for reconstructing hand injuries, while caution is advised for skin, bone, and tendon transplantation, especially in cases of vascular injuries [113]. The successful use of cryopreserved fingertip skin for treating amputations, even in cases where replantation is not possible, has been reported [139]. This approach may serve as a new treatment option after fingertip amputation. However, for extensive resections, both biological and prosthetic materials have not yielded favorable outcomes [130].

### 2.16. Skin

Skin grafts are primarily used for burn wounds, skin disorders, and reconstructive plastic surgery [140,141], with allografts sometimes permanently covering wounds without rejection [142]. Allograft skin is usually used as temporary wound coverage [142], while autologous skin is considered the treatment of choice for permanent wound closure [140]. Cryopreserved skin is also effective in treating extensive Hidradenitis Suppurativa, showing minimal contraction and no disease recurrence [143].

While allogeneic skin grafts, xenogeneic materials, and body contour surgery results can serve as donor tissues [141,144], logistical difficulties and legal frameworks complicate allograft use [144]. Different storage modalities, like refrigeration, cryopreservation, lyophilization, and glycerol preservation, exist. The cryopreservation of skin allografts requires equilibration in DMSO or glycerol and storage in LN or a freezer.

Cryopreservation maintains viability [141] but requires refrigeration within 18 h of death. Glycerol preservation, though termed non-viable, is simpler and more cost-effective, making it preferable in most Western European burn centers, while cryopreservation is more common in the USA. Glycerol-preserved allografts are reported to be superior for children, the elderly, and in sandwich grafting techniques [144].

### 2.17. Brain and Nerve

Brain cryopreservation is mainly promising for research purposes and cryonics. In cryonics, brain cryopreservation is progressively becoming the method of choice over full-body preservation due to the simplicity and cheapness of the method. A group of scientists focused on bridging an interventive procedure with animal experimentation, showing that cryopreservation in DMSO and glycerol preserved the density of synaptic connections and neuron numbers [145].

Research on brain tissue can also be developed with an appropriate cryopreservation protocol that would not require immediate brain tissue dissociation of postmortem animal models or postoperative human samples. In samples of 1–3 mm preserved brain, it was demonstrated that neuronal intact cells survived cryopreservation and the dissociation procedure for sucrose and protease inhibitors [146], which indicates that a large number of living cells can migrate from the tissue on day 14 for a cocktail of methylcellulose, ethylene glycol, DMSO, and Y27632 [147]. The latter technique has also been proven applicable to brain tissue organoids, demonstrating no disruption of the neural cytoarchitecture or functional activity.

Nerve preservation and transplantation techniques are essential for the treatment of severe trauma to extremities. Xenogeneic nerve transplants for large-gap peripheral nerve injuries show regeneration of the transected nerve and, importantly, recovery of wrist extension function, distal muscle reinnervation, and recovery of nerve conduction velocities, but increased inflammation [148].

### 2.18. Muscle or Groin Flap

The most common methods for muscle or groin flap preservation are directional freezing and vitrification [149]. Those methods can maintain composite tissue over a long period in a state adequate for transfer, without loss of viability. However, a study that demonstrated the viability of cryopreserved complex tissue, including rat muscle flaps, compared to artery and vein grafts, based on a technique of whole animal perfusion, showed severe endothelial cell damage with extensive sloughing in the grafts [150].

Technologies such as ex vivo perfusion and subzero preservation demonstrate the potential to extend the allowed time between solid organ procurement and transplantation. Therefore, a two-stage vitrification-based approach for long-term cryopreservation of composite vascularized tissues was demonstrated to ultimately enable their prolonged survival following syngeneic/autologous retransplantation. The long-term survival of below-the-knee limbs cryopreserved by this protocol was significantly superior to that of limbs cryopreserved by directional freezing or cooled to temperatures below the vitrification solution (20% failure compared to 100%) [149].

### 2.19. Other Tissue

Lung cryopreservation, although discussed in reviews [151], faces challenges due to its complex cellular composition, with limited results reported.

The current state of organ and tissue cryopreservation is reflected in Table 1. The abbreviations for CPAs are listed at the end of the table. CPAs containing more than six elements are not detailed in this table (Supplementary Materials).

## 3. Biobanking

Biobanking processes and stores cryopreserved tissues and organs, serving as hubs for the storage and study of biological samples. The Biobanking and Biomolecular Resources Research Infrastructure—European Research Infrastructure Consortium directory lists 432 biobanks as of 1 June 2024 [167]. The first research biobank in Russia, established in 1998, studied thyroid tumors post-Chernobyl. In 2019, the Russian National Association of Biobanks and Biobanking Specialists (NASBIO) was founded to implement international standards in Russian biobanking.

The Sechenov University Biobank, established in 2017, is part of the research medical facility with the largest clinical center in Russia and Eastern Europe. The biobank serves more than 550,000 patients per year. It includes a diverse collection of samples and covers various pathologies, which supports a wide range of research projects. Through its advanced facilities and comprehensive sample collection, the Sechenov University Biobank significantly advances scientific knowledge and innovation in the biomedical field. The Sechenov University Biobank collection includes more than 150,000 samples of biological fluids (serum, plasma, urine, saliva, cerebrospinal fluid), various cell cultures, and human gut microbiota samples.

**Figure 2:**
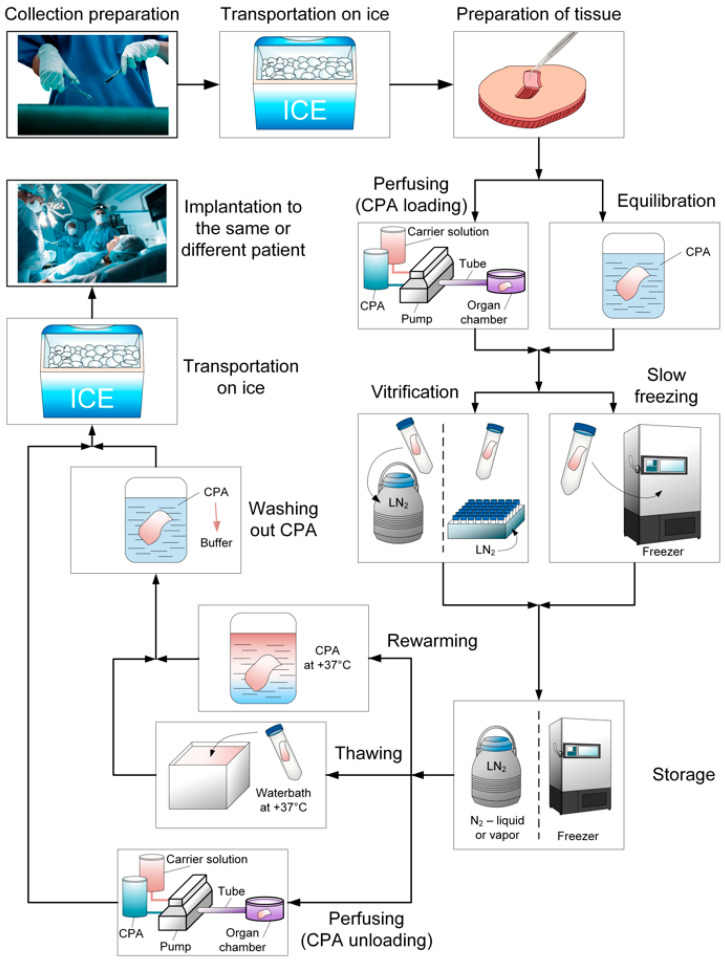
The main steps of the tissue and organ biobanking process.

Despite current limitations, promising results in tissue and organ cryopreservation, particularly ovarian tissue, have been achieved. Figure 2 outlines the biobanking process.

## 4. Main Features and Parameters of Cryopreservation

### 4.1. Cryopreservation Methods

Cryopreservation methods typically involve slow freezing and vitrification techniques. Some classifications also include static cold storage, primarily used in clinical organ preservation. Static cold storage reduces organ metabolic rates by maintaining them at 4 °C for several hours [110].

#### 4.1.1. Slow Freezing

Cryopreservation preserves cells in the presence of ice crystals, ensuring viability during freezing and thawing. Slow freezing initiates ice crystal growth outside the cell, creating an osmotic gradient that expels water from the cell. This reduces intracellular water, minimizing ice formation inside the cell. While extracellular ice is less harmful, intracellular ice can disrupt cell structures, making an understanding of these dynamics crucial for optimizing techniques [6,168].

During cryopreservation, cellular dehydration elevates intracellular electrolyte concentrations, preventing ice formation but causing osmotic stress and potential solute damage [6,168]. The optimal cooling rate balances slow freezing and vitrification techniques to prevent cryoinjury from solution effects [8] and intracellular ice formation. Colligative cryoprotectants help safeguard cells by preventing harmful volume changes and inhibiting ice formation [168].

To overcome the issues with lowered viability due to a fast cooling rate, researchers usually prefer to use slow cooling protocols with a rate of cooling around 1 °C per minute and less than 1.0 M of a cryoprotective agent [6,8,169,170,171]. Controlled slow freezing, gradually cooling cells to −196 °C, is standard, while uncontrolled freezing is less common [166]. Slow freezing results in less than 30% cell loss [172], but efficient protocols for tissues and organs are limited to small sample types.

#### 4.1.2. Vitrification

Cryopreservation in a vitrified state avoids ice crystal formation by solidifying samples in a glass-like state during cooling and rewarming, differing from freezing/thawing methods. Vitrification involves transforming cell suspensions to a vitreous state using liquid nitrogen (LN) or slush at temperatures between −80 °C and −130 °C.

This process requires increasing the viscosity of the medium and cells to prevent ice formation, maintaining stability during storage at the glass transition temperature. Vitrification can be achieved by directly immersing the cryovial into liquid nitrogen [168]. Cryoprotective agents (CPAs) and dehydration increase viscosity, facilitating the glass-like state and preserving molecular integrity indefinitely [6,171,173]. Vitrification uses higher CPA concentrations (30–50%) compared to slow freezing (5–10%), with cooling and warming rates achievable in laboratory conditions.

However, high CPA concentrations can harm cells, causing osmotic injury and toxicity. Section 6.5 discusses methods for mitigating CPA toxicity, including combining different CPAs. Osmotic damage is associated with the magnitude of volume excursion, exposure duration, and temperatures during CPA addition/removal [174]. Vitrification success depends on sample viscosity, cooling/warming rates, and sample volume [171]. Controlling viscosity involves high CPA concentrations and water removal via osmotic dehydration or evaporative desiccation [168].

Direct contact cooling achieves high rates but risks pathogen transmission and contamination. Closed vitrification systems offer lower cooling rates but require higher CPA concentrations [175]. Optimizing rewarming is crucial for stabilizing the glass state and preventing fractures and ice crystal formation during devitrification [168].

The comparison of these methods is summarized in Table 2 [6,171].

### 4.2. Cryoprotectants

Cryoprotection is crucial in cryopreservation, involving the addition of CPAs to minimize ice crystal formation, depending on cell type, cooling rate, and warming rate. CPAs are classified as membrane-permeating or non-permeating [10,171,172,176]:

- Permeating: dimethyl sulfoxide (DMSO), glycerol (GLY), 1,2-propanediol (PrOH), ethylene glycol (EG), methanol.
- Non-permeating: polymers (polyvinylpyrrolidone, hydroxyethyl starch, polyethylene glycol) and sugars (raffinose, sucrose, trehalose, glucose, mannitol, galactose).

In vitrification, CPAs prevent ice formation by lowering the freezing point and increasing viscosity and the glass transition temperature [6].

Slow freezing removes water to avoid intracellular ice, using permeating CPAs like DMSO to raise the glass transition temperature and reduce the freezing point [173]. CPAs must be removed after thawing to prevent toxicity. Colligative cryoprotectants, combined with osmotic additives (polyols, sugars), enhance cell recovery during thawing by removing freezable water [168,177].

While glycerol and DMSO are commonly used, their toxicity requires thorough washing during the thawing process [173]. Various other types of cryoprotectants can also be implemented to preserve biological tissue [66].

## 5. Sperm Cryopreservation

Cryopreservation of cells, tissues, and organs is widely used in biomedicine and scientific research, particularly for molecular genetic screening. For most clinically significant cell cultures, tissues, and biological materials, separate safety studies are conducted. Investigating the impact of cryopreservation on the safety of biological materials intended for future clinical use is a critical issue that requires thorough examination.

Currently, there are three main methods for long-term sperm cryopreservation: slow freezing (stepwise controlled cooling to a temperature of −80 °C over 2–4 h, followed by transfer to liquid nitrogen for storage) [178], rapid freezing (freezing for 8–10 min in nitrogen vapor, then transferring to liquid nitrogen), and vitrification. These protocols differ not only in freezing and thawing rates but also in the volume of cryoprotective media added to the sperm [179].

Unlike oocytes, whose preservation and survival remain a challenging task [180], sperm cryopreservation has long been a routine process and is successfully used to preserve fertile spermatozoa in various animal species and humans [181,182]. Since the shape, size, composition, and organelle structure vary significantly across species, cryopreservation protocols are adapted according to these differences. However, even with careful optimization of conditions, approximately 40–50% of sperm do not survive the cryopreservation process [183,184]. For example, the survival of horse sperm depends not only on the freezing rate but also on the season of material collection, while the quality of frozen bull and cattle sperm is comparable to that of freshly collected samples [184].

The processes of freezing and subsequent thawing of sperm have a destructive effect on various cellular functions, typically leading to reduced motility and viability, decreased mitochondrial activity, disruption of DNA integrity, and increased production of reactive oxygen species (ROS), ultimately impairing fertilization capacity [179,185].

The cryoprotective media used for mammalian sperm typically consists of penetrating cryoprotectants such as glycerol and DMSO, combined with non-penetrating cryoprotectants like albumin, dextran, egg yolk, and pasteurized milk [186]. The addition of various antioxidants to the sperm cryopreservation medium, such as vitamin E, glutathione (GSH), sericin, superoxide dismutase (SOD), catalase (CAT), vitamin C, melatonin (MLT), and selenium (Se), helps reduce oxidative processes and the formation of free radicals [185,186,187].

Below is the impact and use of the most common types of cryoprotectants in sperm cryopreservation (Table 3).

## 6. Current Challenges of Cryopreservation

Optimizing cryopreservation protocols is crucial. Despite advancements, effectively preserving various cells, tissues, and organs remains challenging, especially for solid organs and complex tissues, with viability often limited to hours [2]. The first global summit on organ banking [196] addressed several issues. This section elaborates on the current state and potential solutions.

### 6.1. Optimization of Protocols

Cryopreservation stages carry many risks [197], and cryopreservation requires the careful optimization of CPAs, incubation time, and freezing/thawing rates [168,198]. Specific cooling and warming protocols can help avoid devitrification and recrystallization issues [199]. However, implementing such protocols is challenging for tissues with various cell types [6].

Rapid cooling or warming near the glass transition temperature can fracture the glass. The number of freezing/thawing cycles also affects sample stability [168]. Successful cryopreservation depends on the cooling and thawing rates necessary for cell survival [8,177]. In vitrification, CPAs prevent ice formation when cooled faster than their critical cooling rate (CCR). Achieving the critical warming rate (CWR) is more challenging [2]. Slow warming increases the risk of ice formation and cryoinjury. For example, devitrification in vascular allografts can cause artery microfractures and graft rupture [52]. High cooling and warming speeds are difficult to attain in larger samples, leading to thermal gradients, cracks, and ischemia–reperfusion injury [99,108].

Tissue recovery is crucial for optimal cryopreservation. Various compounds are being explored for revival–recovery–repair cocktails to restore full tissue function [2].

### 6.2. Storage and Transfer of Samples

Lower storage temperatures increase sample stability and preservation duration. For biobanks, storing samples below the cryoprotectant’s glass transition temperature ensures long-term stability [8]. Cryogenic storage in liquid nitrogen is standard for preserving viable cells, tissues, and organisms [168]. However, even liquid nitrogen storage has limits, and the lack of standardized evaluation methods for tissue deterioration hampers protocol improvements and the extension of storage times. Standard guidelines, such as those for heart valves [44], have helped double the expiration period for blood vessels and heart valves [49].

Continuous temperature control during storage and transport is crucial. Transferring frozen samples from a controlled-rate freezer to storage or transport containers requires care, as brief exposure to elevated temperatures can cause microscopic melting and compromise viability [8]. Freezing and thawing homograft valves can lead to surface and structural damage [200]. Transfer protocols and temperature changes are often inadequately described, and not all biobanks keep records of thawing or restocking or monitor freezer temperatures [201].

### 6.3. Large Sample Size

Cryopreservation of complex tissues and organs, unlike isolated cells, is challenging due to sample size. For samples larger than a few milliliters, temperature distribution affects cell function and recovery [202]. Larger samples increase the risk of uncontrollable crystallization and temperature nonuniformity, causing thermomechanical stress [196].

Large tissues struggle with rapid cooling and heating rates and uniform CPA distribution due to varied heat and mass transfer properties [21]. Cooling rates differ between the core and surface, with the core cooling more slowly, leading to surface ice nucleation. Additionally, latent heat release during ice formation increases the temperature lag between the rim and core [114]. Ensuring complete CPA penetration before freezing [24] and CPA removal after thawing is crucial to avoid toxicity at physiological temperatures.

Ice crystallization in tissues and organs is harder to manage with traditional cryoprotectants and controlled cooling/thawing rates, leading to cellular damage. Unregulated ice formation during cryopreservation remains a significant barrier to tissue survival through freezing and thawing [2].

A potential solution is nanowarming. Inspired by biologically regulated thawing, researchers have developed electromagnetic [203] and magnetic nanoparticle warming methods [204]. In this approach, nanoparticle CPAs act as self-heating seeds activated by external fields rather than heat conduction. This method has been effective in porcine arteries and heart valve tissues [113]. Nanowarming accelerates warming rates, reduces CPA amounts, and mitigates temperature nonuniformity [108]. It uses magnetic nanoparticles in an alternating magnetic field for homogeneous and rapid rewarming. However, this method may face challenges in complex tissues like lungs [151].

### 6.4. Cryoinjury

Cells and tissues are usually preserved by slow freezing, which forms ice. Despite advancements, cryopreservation cannot ensure successful tissue revival with ice formation due to cell damage and disrupted cell interactions [2,110,196]. This is critical for samples like corneal endothelium cells, which have limited proliferative capacity [90].

Potential solutions are as follows:

1. Controlled, Partial-Ice Freezing

Inspired by the wood frog (Rana sylvatica), which survives with 65–70% of its body water as extracellular ice, controlled partial-ice freezing aims to minimize cryodamage. This method uses biocompatible ice nucleators for slow freezing at high subzero temperatures. Initial success has been shown in blood vessel models [2] and might be beneficial for complex tissues like lung tissue [151].

2. Directional freezing

Directional freezing uses a temperature gradient to induce progressive ice growth, either by moving the sample or placing it between thermo-conductive blocks. This results in homogeneous cooling [25], forming ice lamellae that trap cells and reduce mechanical damage [6,112]. Directional freezing is key for long-term whole-organ cryopreservation [30,205] and is effective for complex tissues [137]. The further goal of directional freezing, according to researchers, aims to lower organ freezing temperatures to −130 °C [6].

3. Machine perfusion

Machine perfusion technology enhances CPA delivery and washing, crucial for vascularized tissues and organs. It plays a vital role in preconditioning, preservation, and recovery stages, making it pivotal for innovative cryopreservation approaches [38,99,100,102,112].

### 6.5. CPA Toxicity

CPA toxicity is a significant challenge in cryopreservation. Toxicity mechanisms are either specific (unique to a particular CPA, such as ethylene glycol (EG) causing metabolic acidosis) or non-specific (shared properties, such as hydrogen bonds denaturing proteins at high concentrations) [6]. CPA administration and removal introduce mechanical stresses, reducing success rates, particularly in vitrification [5]. The viscosity of vitrification solutions limits their use in larger samples. Lowering temperatures can reduce toxicity, but increased viscosity restricts loading temperatures [2].

Vitrification involves exposing cells or tissues to high CPA concentrations (40–60%, mass/volume) and rapidly cooling to deep cryogenic temperatures using LN to prevent ice nucleation [171]. High CPA concentrations are necessary to replace water and lower the CCR [6].

Potential solutions are as follows:

1. Liquidus Tracking

Due to the harmful effects of high concentrations of CPAs [171], an alternative cryopreservation strategy could potentially be used for more sensitive or larger samples. Liquidus tracking involves stepwise CPA delivery during cooling, keeping the system above the equilibrium freezing point, thereby preventing ice crystal formation and enabling vitrification without ultra-rapid cooling [2,168]. Gradual CPA addition reduces toxicity, while stepwise removal during thawing minimizes osmotic stress [206]. However, this method requires further development [6,144,207].

2. Neutralizing toxicity

Combining different CPAs, such as sugars with low concentrations of DMSO, can reduce toxicity [6,172]. Certain CPAs neutralize each other’s toxic effects, allowing for higher total concentrations with reduced overall toxicity. For example, combining DMSO with formamide [6], or replacing some DMSO with glycerol or ethylene glycol, enables vitrification with significantly less membrane damage [208].

3. Antifreeze proteins and (glyco)proteins as ice-binding proteins

Antifreeze proteins and glycoproteins (AF(G)Ps) bind to ice, preventing its growth without significantly lowering the equilibrium freezing temperature. They adsorb onto ice crystal surfaces, inhibiting growth within a specific temperature range when their concentrations are sufficiently high [209]. Despite their effectiveness, challenges such as extraction, immunogenicity, cost, and the formation of needle-shaped ice crystals limit their broader application [176,210]. Further cooling beyond moderate temperatures can cause uncontrolled crystal growth, potentially leading to damage [173]. Glycolipids also protect against freeze damage by binding to nucleation sites or stabilizing cell membranes, particularly when combined with glucose [173].

4. Synthetic polymers

Current research focuses on designing synthetic ice modulators to mimic AF(G)Ps. Synthetic polymers, such as polyvinyl alcohol (PVA) and polyglycerol (PG), act as non-penetrating cryoprotective agents (npCPAs) by inhibiting ice recrystallization or growth. PVA prevents crystal enlargement, while PG inhibits ice formation. These alternatives to animal-derived antifreeze proteins are cost-effective, non-toxic, and effective across a wide temperature range [6,210]. Mimicking the 3D structure of AF(G)Ps offers another protective strategy for cryopreservation, with low cytotoxicity and suitability for large-scale production [110,176]. Synthetic polymers can be assembled from various monomers, providing flexible structures and functions. Ice recrystallization inhibitors (IRIs) are considered potential additives to traditional CPAs [211]. However, synthetic IRIs are currently less effective than their natural counterparts [209], and ongoing research aims to enhance their potency, reduce toxicity, and improve stability [2].

5. Natural low-molecular-weight CPAs

Another category of non-penetrating cryoprotective agents (npCPAs) includes small molecules like sugars, such as the non-metabolizable glucose derivative 3-OMG, trehalose, L-proline, β-alanine, γ-aminobutyric acid, and ε-aminocaproic acid. These molecules have gained attention for their ice inhibition properties [2,110,173,176]. Their effects differ from those of DMSO, as cold-tolerant animals accumulate various natural CPAs like L-proline and trehalose to survive low temperatures [212]. NpCPAs are less toxic and require lower concentrations for effective cryopreservation [2,173]. They function by dehydrating cells, protecting membranes by lowering phase transition temperatures, and inhibiting intracellular ice formation. NpCPAs can also be used in thawing media to prevent osmotic shock and cell lysis [173]. Using npCPAs as additives enables the use of lower concentrations of more toxic penetrating CPAs, improving cryopreservation outcomes [6]. However, penetrating CPAs remain the most promising option for tissue and organ cryopreservation [173].

6. Permeation for npCPAs

Cold-resistant species use sugars like trehalose and sucrose as natural CPAs, but their natural intracellular production limits their use as artificial npCPAs due to their inability to enter cells where they would have an effect [110,171]. Efforts to modify these sugars for permeability while retaining cryoprotective activity have been explored. For non-permeable CPAs, nanoparticles designed to carry CPAs into cells and release trehalose in response to pH triggers have been developed [213]. This approach serves as a reference for the intracellular delivery of other non-penetrating bioinspired CPAs.

7. Ice nucleators

Organisms survive extreme cold by producing ice nucleators, which promote controlled freezing of extracellular water at multiple sites. This allows freezing at relatively high subzero temperatures before intracellular freezing occurs, minimizing cryoinjury [2]. Ice nucleators include cholesterol, silver nitrate, silver iodide, ammonium sulfate, bacterial proteins, minerals, alcohols, nanoparticles, and physical induction methods like ultrasound or laser exposure [6,8]. While this approach is simple, precise timing for the addition of ice nucleators is crucial to prevent excessive dehydration or uncontrolled ice growth [110]. For smaller samples, nucleation can be induced through rapid cooling, followed by returning to the original temperature, close to the melting point of the suspending medium [67,69,152,166,214].

8. Non-Newtonian and Rheomagnetic Fluids

Non-Newtonian methods have been proposed to expedite CPA loading and unloading, reducing diffusion time and temperature to enhance post-preservation outcomes. By varying shear force after loading, a non-Newtonian pseudo-vitrification state can be achieved during cooling to −60/−70 °C. This approach increases viscosity, reducing toxicity and inhibiting ice formation while allowing for lower CPA concentrations. External magnetic fields can also alter viscosity using the rheomagnetic pseudo-vitrification method [2].

9. Directional vitrification

A technique related to directional freezing is directional vitrification; this is achieved using the same principle as directional freezing but with a very high cooling rate [6]. Both directional freezing and directional vitrification offer promising avenues for future research. However, information on directional vitrification is limited.

10. Isochoric cryopreservation, thermodynamic equilibrium pressure-enabled cryopreservation

High-subzero isochoric preservation offers more thermodynamic flexibility than conventional constant-pressure cryopreservation [215]. In this method, a pressure-resistant constant-volume chamber prevents volumetric expansion from ice formation, limiting ice growth while maintaining necessary pressure levels [2,172]. Ice crystal growth occurs separately from the biological sample within the chamber, keeping the sample ice-free at temperatures well below freezing and atmospheric pressure. This approach offers benefits such as ice-free preservation down to −20 °C, minimal CPA concentration or none at all, and suitability for tissues and organs [2]. Isochoric conditions can enhance the probability of vitrification, reducing the likelihood of random ice nucleation and requiring lower concentrations of chemical additives [2,110].

## 7. Conclusions

In summary, the study of cryopreservation methods for various tissues and organs reveals a dynamic and evolving field critical for advancements in medicine. Vitrification and slow freezing remain the two primary techniques, each suitable for different types of tissues and organs depending on their cellular composition and functional requirements. Innovations in cryoprotective agents (CPAs) and cooling protocols have significantly improved survival rates and functionality post-thaw. However, issues such as CPA toxicity, ice crystal formation, and thermal stress continue to pose significant challenges.

For tissues routinely used in clinical practice or biobanking, we could highlight ovarian tissue, immature testicular tissue, heart valves, vascular tissue, adipose tissue, bone, and skin. Ovarian tissue cryopreservation is a well-established technique and has been proven by existing live births after retransplantation. However, freezing damage and ischemic issues hinder whole-ovary preservation. Immature testicular tissue cryopreservation is offered in biobanks but remains experimental due to the lack of reported human live births. Cryopreserved heart valves are a standard biobanking procedure with quite a good rate of survival. Cryopreserved vascular tissue also has standardized protocols for cryopreservation established in many biobanks, showing fairly good survival rates. Cryopreserved adipose tissue is a good option for multiple injection applications with minimal complications. Amniotic membranes can be well preserved at cryogenic temperatures and used for various applications. Autologous and allogenic bone cryopreservation is an established technique, especially for cranioplasty but allogenic material does not yield successful results in a lot of cases. Cryopreservation of skin is used as a viable option for wound closure. It is proposed in many burn centers.

Other tissue and organ types have all yielded some successful results, but for some reason, their use has not been as widespread, usually because of the size of the samples. The heart has not yet shown any signs of a successful cryopreservation technique, with small animal hearts frozen to −30 °C showing that younger organs could sometimes be reanimated. Despite advances, cryopreserved pancreatic islet recovery is low due to damage during thawing and rewarming, making them clinically inapplicable, although pancreatic islet biobanking is accessible in several countries. The corneal endothelium is highly susceptible to cryoinjury, resulting in lower integrity and viability compared to fresh corneas. Therefore, eye banks do not routinely use corneal cryopreservation. The success of kidney cryopreservation is very limited, with some advances in the use of very small animal kidneys with or without nanowarming. Scaling liver cryopreservation to human organs is limited even though there have been some reports of successful animal studies. Different cryopreservation techniques for cartilage are available. However, it is not widely used in biobanks. Cryopreserved tracheal allografts and epithelium-denuded-cryopreserved tracheal allografts have been shown in the literature to be a viable tracheal replacement, but this technique requires further research. There is relatively little successful research on complex tissue, containing several different tissues. Successful cryopreservation of digits has been reported in the literature, but it has not become a widespread technique yet. Brain tissue cryopreservation is becoming a way to save neural tissue for subsequent studies and has shown some promise, and nerve tissue has shown some successful results in animal transplantation. There are some papers on successful muscle and groin flaps demonstrating their viability after cryopreservation.

Emerging technologies, including nanotechnology, offer promising avenues to address these challenges. The development of tailored cryopreservation protocols, informed by a deeper understanding of tissue-specific responses to freezing and thawing processes, is essential. A promising direction of development is the creation of new freezing protocols based on the search for and use of minimally toxic or non-toxic cryoprotectants.

Overall, while substantial progress has been made, the field of cryopreservation is still in its developmental stages. Continued research is imperative to refine these techniques, improve preservation outcomes, and expand their applicability across a wider range of tissues and organs. The ultimate goal remains the establishment of reliable, efficient, and safe cryopreservation protocols that can significantly enhance the prospects of regenerative medicine, organ transplantation, and biobanking.

## Figures and Tables

**Table 1:** Current state of tissue and organ cryopreservation.

Tissue/Cryopreservation Method	Object	CPA	Sample Size	Results	Reference
**Ovarian Tissue**					
SF	Human	PrOH/SUC/ALB	1 mm	85% of intact oocytes and 74% of intact pre-granulosa cells. Preservation of function assessed by xenografting and longevity—in 1 human patient over 5 years.	[152]
V	Human	EG/DMSO/SSS + SUC	10 × 10 × 1 mm^3^	Hormonal return by 150 days and menstruation by 130 days. 92% viability of oocytes and 5 live births.	[23,24]
Mostly SF	Human	-	-	43.8% clinical pregnancy rate, 32.3% live birth rate, 7.5% miscarriage rate.	[153]
Mostly SF	Human	-	-	57% live birth rate.	[154]
DF	Sheep	FBS/DMSO	Whole organ	Results similar to fresh organ and higher than slow freezing: 89% intact follicles; 17,041 nuclei/mm^2^ stromal cell density; intact vessels.	[30]
Not specified	Human	-	Slices	33.84% successful in vitro maturation of immature oocytes. 50–76.9% fertilization rate.	[155]
**Testicular Tissue**					
CSF	Mouse	DMSO/SUC	<1.8 mL	Similar to control: 47.6 ± 19.2 donor-derived colony numbers and 13.4 ± 7.2 × 10^4^ cell recovery. Worse than control: 83 ± 2% post-thaw cell viability and 6.2 ± 0.2 pups per litter.	[156]
USF	Human	DMSO/SUC/HSA	3 mm^3^	Similar to control: structural integrity/morphology; intact seminiferous tubules; SSCs and Sertoli cells.	[157]
USF	Mice	DMSO/SUC	-	Similar to control: cellular integrity; 92% graft survival. Lower than control: 77 ± 12% intact tubules.	[35]
CSF	Human	DMSO/SUC/HSA	3 mm^3^	Similar to control: structural integrity/morphology; Sertoli cells; intact seminiferous tubules. Higher than control: SSCs with number of OCT4-stained cells.	[157]
V	Human	DMSO/EG/HSA	3 mm^3^	Similar to control: structural integrity/morphology; SSCs and Sertoli cells; intact seminiferous tubules.	[157]
Solid-surface V	Mice	DMSO/EG	3 mm^3^	Similar to control: 75% graft survival; 157 ± 109% of tubules completed spermatogenesis. Worse than control: 24 ± 22% damaged SSCs; 75 ± 9% intact tubules.	[35]
**Heart**					
CSF and USF	Wistar Rats	DMSO, O_2_, CO_2_	Whole organ	10–15% successful reanimation. Always could be reanimated, if supercooling occurred. Below freezing point, resuscitation impossible. 10–18-days-old animals survived after −30 °C.	[38]
USF	Minipigs	Skinning solution	~0.5 cm^3^	Similar to control: myofilament lattice arrangements; structural integrity; contractility.	[41]
**Heart Valve**					
CSF	Human	DMSO	-	Infective endocarditis: 47% 3-year survival rates; 76% freedom from re-operation. Non-infectious heart disease: 1 case of hemothorax; no graft-related complications.	[42,158]
CSF and V	Pig	VS55, VS49, or DP6/DIS/SUC/TRE	-	Muscle and leaflet viability were the same after vitrification and nanowarming. Increasing the CPA loading duration increased aorta viability after vitrification.	[159]
CSF	Human	DMSO	-	Similar to control: Young’s modulus and thickness. For AAHV, Young’s modulus is higher if stored <5 years; for PAHV, lower.	[49]
Not specified	Human	-	-	Mortality: 2.8% (nonendocarditis group) and 7.2% (endocarditis group). Probability of infection: 5.6%/14% (20 years) in the nonendocarditis/endocarditis groups.	[48]
**Vascular Tissue**					
CSF	Human	DMSO	5 cm	Used for reconstruction of the middle hepatic vein branches, hepatobiliary and vascular surgeries.	[52]
Not specified	Human	DMSO	-	Commercial protocol. No available data.	[60]
CSF	Human	HA/DMSO	-	Similar to control: 1.9% vascular complications. 1-year survival rate: 100%. Higher than control: 5.6% intraoperative complications. Lower: 37% postoperative.	[60]
CSF	Human	DMSO/HA	-	Early postoperative complications: 50.7%; survival rates at 5 years: 54%. Early postoperative reintervention: 15.5%; late reinterventions: 18.3%.	[56,160]
**Pancreatic Islet**					
V	Mouse, Pig, Human	EG/DMSO	-	Similar to control: 87.4% islet viability in humans at 9 months. 96.7 ± 1.5% islet recovery in humans; 92% restored normoglycemia within 24–48 h in mice.	[67]
CSF	Mouse, Pig, Human	DMSO	-	59.1–62.2% islet viability (lower than vitrification).	[67]
Not specified	Human	Islet cryopreservation solution	0.075 mm^2^	Volume change was only reduced by oxygenation in the long-term recovery rate. The oxygenated method suppressed ischemia- and inflammation-related gene upregulation.	[68]
CSF	Human, Mice, Rat	DMSO	-	Better with EG: 80.4 ± 2.6% yield, 80 ± 1.5% viability, and 3.4 ± 0.4 glucose-stimulated insulin release after 48-h culture in humans; euglycemia achieved 12 days sooner.	[69]
V	Rat	EG/DMSO/FBS + SUC	0.03 mm^2^	Lower than control: viability. Similar to control: stimulation index and response to glucose uptake. 80.0–83.3% euglycemia in 3 weeks; 93.8–97.1% recovery rate.	[70]
**Adipose tissue**					
CSF	Human	DMSO/TRE or TRE	-	Less functional (G3PDH), even with no obvious differences. SVF = 90% of control. Similar to control: cell viability at 2 weeks.	[77]
CSF	Human	Not precise	4 mL	Similar to control: histology. No complications for all patients. Higher than control: 14.8 × 10^5^/mL SVF.	[72]
USF	Human		10 mL	43.1 ± 7.2% of initial volume preserved at 6 months. Complications: 9 infections; 7 formations of nodules; 3 hematomas; 3 traumatic fat necroses.	[78]
USF	Human		10 mL	91% of initial volume preserved at 12 months. The least favorable results were for leg and lip. Complications: 7 formations of nodules; 3 hematomas; 3 traumatic fat necroses.	[73]
CSF	Human	DMSO/HA	50 mL	Similar to control: 65.7–86.7% cell viability (not affected by subsequent temporary storage at −80 °C); expression of specific membrane markers; adipogenic abilities.	[161]
**Amniotic Membrane**					
USF	Human	GLY	7.6 cm × 1.9 cm	Similar to control: % of degenerated cells without CPA, integrity ± CPA, tensile strength, and Young’s modulus. Worse than control: % of degenerated cells with CPA; cell viability.	[85]
USF	Human	GLY/RPMI or GLY/DMEM	3.8 × 0.7 cm^2^ [86] 4 × 4 cm^2^ [162]	Similar to control: strain at break, resorption behavior [86], protein and growth factor levels [162]. Worse than control: maximum force [86]. Epithelial cells slightly damaged by cryopreservation.	[86,162]
CSF	Human	DMSO/FBS	25 mm^2^	Similar to control: levels of pluripotency gene expression. Worse than control: metabolic activity, significantly.	[163]
CSF	Human	GLY or DMSO	-	-	[164]
**Cornea**					
CSF	Human	HSA/DMSO (#1); DMSO (#2); VS55 (#3); EC/PrG/DMSO (#4).	1 cm^2^	Worse than control: all cell monolayers disrupted; cell density decreased; disruption in the collagen matrix of stroma (#1, 3, 4 CPA); epithelia flattened (#1, 2); separation between endothelia and Bowman’s layers (2); endothelia not visible in central part (#3, 4).	[91]
Not specified	Human	Eusol-C	1 cm^2^	76% of corneas achieved epithelization; 50% were clear; 38% developed neovascularization. Complications: 34% reintervention; 8% positive microbial cultures; 4% corneal abscess.	[92]
**Kidney**					
V	Rat	EC/DMSO/FA/EG/X-1000/Z-1000	Whole organ	After transplantation, kidneys reperfused immediately and uniformly, making urine in 2 min.	[102]
V, Nano	Rat	EC + VS55 + mNPs	5.5 × 4.5 × 1.5 cm	Cooling faster than CCR at −45 to −90 °C and rewarming faster than CWR; intact morphology. Good viability; increased red cell death in the glomeruli; vascular endothelial lining intact.	[99]
V, Nano	Rat	VS55	<5 mL	Better than bath thawing: maximum thermal stress, flow resistance. Rewarming faster than CWR. Internal renal cell structure slightly damaged; vascular network slightly injured; 38 ± 8% renal cell apoptosis.	[100]
V	Rabbit (Embryonic Kidneys)	EG/DMSO/N-MET/MP/POLY/X-1000 or Z-1000/FBS	-	35% metanephroi from 16-day-old embryos were successfully grown, underwent differentiation, and developed histologically mature glomeruli. Histomorphometry similar to nonfrozen.	[103]
V	Microminiature Pig (Embryonic Kidneys)	FBS/EG/DMSO	1.37 × 0.90 mm^2^	Kidneys increased in size and reddish at 14 days. 4 glomeruli per slide and sparse medulla, similar to native neonatal. Presence of glomeruli, proximal and distal tubules, and collecting ducts.	[104]
**Liver**					
V, Nano	Rat	EG/sucrose/EC + mNPs	<8 × 13 cm^2^	Complete removal of the mNPs and CPA. Temperature gradient below cracking. Intact cellular and sinusoidal architecture, preserved endothelium and hepatocyte function, and homogeneous perfusion. ALT levels slightly higher than control; LDH levels similar to control.	[109]
DF	Rat, Pig	UW/EG	Whole organ	Pig: 75–90% viability; satisfactory reperfusion, no blood flow obstructions. Warming up with homogeneous color within a few minutes; slow bilious-stained secretion.	[112]
CSF	Murine	EDTA+ UW/EG	Whole liver	Less than control: 60 ± 15% bile production (1) and 84 ± 17% (2); 63 ± 11% integrity of hepatocyte cell (1) and 85 ± 6% (2). Similar to control: tissue architecture. Normal presence of the epitope.	[112]
CSF	Pig	UW/EG	Whole liver	Viability ranging from 75% to 90%. Transplanted liver was functional.	[112]
**Cartilage**					
V	Pig	VS83,VS70 or VS55	30 × 20 × 14 mm	Better 1 than 2: no crack formation; cell metabolic activity; cell viability; live cells. Similar among 1, 2, control: conductivities; tissue porosity; permeability.	[116]
CSF and V	Pig	DMSO (CSF); EG/DMSO/CS/PrOH/SUC(V)	Ø7 mm	Similar among CSF, V, control: peak modulus. Higher in V and control than CSF: equilibrium modulus and relaxation time constants.	[115]
USF and V	Pig	-(USF) DMSO/EG/PrOH(V)	Ø10 mm	Similar among USF, V, control: peak stress; secant modulus; equilibrium stress values and Young’s modulus. Different from control: USF and V in stress relaxation time constants.	[114]
**Bone**					
USF	Mini-Pig	-	3.5 cm	Worse than control: 616 ± 67 bone mineral density; TID, IDI, AvgCID, AvgED higher; compressive modulus. 50–87% healed tibiae.	[121]
USF	Mini-Pig	-	3.5 cm	Higher in group with growth factors: healing, periosteal bridging, callus remodeling and union, intramedullary and cortical vascular volumes. 31% cutaneous vascular tumors.	[123]
USF	Human	-	-	Postcranioplasty infections in 12% of patients with reimplantation. 1-year mortality rate in reimplantation group: 16%.	[122]
USF	Human	DMSO	250–1000 µm	Better than control: 35.18 ± 4.98 mm^3^ volume gain, collagen and osteopontin expression. Worse than control: 0.63 ± 0.03 mg/cm^3^ bone mineral density, 46.25 ± 4.36 percent bone volume.	[124]
USF	Human		0.5 × 0.5 cm	Similar for 1 and 2: 1.61% vs. 2.34% PTH1 positive cells. Better 1 than 2: 6.91% OPG-positive cells. Avital/total tissue surface: 2.51% (#1) vs. 0.03% (#2).	[165]
**Trachea**					
USF	Pig	DMSO	2.5–3.0 cm	The shortest/longest survival period was 1 day/147 days. Average 61.8 days. Fused tissue was formed between the allograft aorta and tracheal cartilage of the original trachea.	[133]
CSF	Pig	DMSO, HSA	5 cm	Similar to control: shape, compression, structure, cytoplasmic granules, GAGs, collagen. Less than control: DNA content, immunoreactivity, elastic fiber pattern and content. Cartilagineous compartment intact and cellular elements at the epithelium side at implantation.	[129]
CSF	Rabbit	DMSO	~6 cm	19.6 ± 16.7 survival time. Satisfactory stiffness. Varying levels of neoangiogenesis and inflammatory infiltration.	[131]
CSF	Rat	FBS/DMSO/SUC	-	Similar to control: chondrocyte viability; at implantation: obliteration, cartilage necrosis, epithelium loss, abundant fibroblasts, macrophage infiltration, and significant immunorejection.	[132]
**Complex**					
DF	Rat	FBS/DMSO/sucrose	Whole limb	Greatest cell viability in 10% DMSO (88.8 ± 0.9%). Cell necrosis and debris increased without cryoprotectant (88.0 ± 6.2%).	[136]
DF	Rat	DMSO/FBS/TRE	Whole limb	Limbs had viable muscle, skin, and blood vessels. Histological structure, nuclei were preserved. Cell size was uniform, and there was no evidence of extreme inflammation.	[137]
V	Rat	DMSO/EG/TRE	Whole limb	Limbs had viable muscle, skin, and blood vessels. Histological structure, nuclei were preserved. Cell size was uniform, and there was no evidence of extreme inflammation.	[137]
**Skin**					
CSF	Human	DMSO	4 × 15 cm	No dead cells or tissue observed over the period of 21 days.	[141]
Not specified	Human	DMSO	1 mm × 3–8 cm	Tissue viability decreased significantly after freezing.	[140]
Not specified	Human	DMSO	25 × 5 cm × 0.5 mm	Intact layers of epidermal tissue.	[142]
Not specified	Human	-	-	99% of graft over the wound at 1 week. Wound contracture, granulation tissue and improved contour after 2nd allograft. Grafts well incorporated with no disease recurrence at 1 year.	[143]
**Brain and nerve**					
USF	Human	DMSO/GLY	Whole brain	Tissue appeared significantly softened	[145]
USF	Rat	DMSO/GLY	Whole brain	No significant differences were detected among groups in brain weight and dimensions, thickness of the cortex and hippocampus, and cell diameter of NeuNir neurons. Mature neuron numbers were comparable; neuron diameter revealed a significant shrinkage. Well-preserved density of synaptic connections. DCX neurons showed a significant shrinkage. No significant changes in TH cell diameter.	[145]
CSF	Mice	FBS/DMSO	0.6–0.7 cm in length	Neurite outgrowth and morphology were comparable to control. Successful isolation of TG neuron cells. No apparent difference in the length of neurite outgrowth; slightly thinner neurites in cryopreserved group. Comparable length and morphology of typical long thread-like axon and short branched dendrite to control. Comparable results in the following parameters: number of neuronal bodies, total neurite length, number of neurite nodes, and number of neurites.	[166]
USF	Human	Methylcellulose, EG, DMSO, Y27632	-	Apoptotic cells reduced in methylcellulose-, EG-, and PVP- treated organoids. TRE-, SUC-, glucose-, and proline-treated organoids showed a larger number of dead cells. Almost all cells in DMSO apoptotic. Functional cytoarchitectures of cortical organoids well preserved. Cell populations, gene expression, or cell spatial distribution of cortical organoids not disrupted. Glutamatergic synapse connections maintained, survival and neural function of organoids maintained. Cell diversity and structure in multiple brain-region-specific organoids preserved.	[147]
CSF	Pig	-	4 cm	No adverse events. Functional recovery equivalent between xenogeneic and autologous limbs for fresh and frozen. Partial remyelination of fast-conducting fibers (autologous and xenogeneic). For xenogeneic transplants, little to no myelination was observed. Median sensory nerve conduction velocity did not return to preoperative baseline levels. Sensory nerve conduction velocity reduced after operation. A nearly complete loss of action potential was observed in all limbs. Maximum recovery was observed 8 months postoperatively. No major qualitative differences were observed in xenogeneic transplant vs. autograft electrophysiology. White blood cell counts and individual component percentages remained within normal ranges following nerve reconstruction with xenogeneic nerve transplant for the entirety of the study (12 months). Xenogeneic transplantation sites had a greater degree of inflammation. No residual porcine tissue in the xenogeneic nerve tissue.	[148]
**Muscle or groin flap**					
CSF	Rat	DMSO/GLY/glucose	4.5 × 4.5 cm	All groin cutaneous flaps, gracilis muscle flaps were necrotic at 3 to 5 days postoperatively. Microscopic evidence of edema, red cell extravasation, and fibrin clot formation in the vascular vessels of groin cutaneous flaps.	[150]
V and CSF	Rat	DMSO/EG/Trehalose/HSA(V)	~10 × 4 × 3 cm	Blood vessel damage after >10 s in LN slush and 2 min in LN vapor. Reduced survival of cryopreserved for 8–9 days compared to flaps cryopreserved for 24–48 h (protocol: two-step vitrification, cryopreservation in −80 °C for 24–48 h or 8–9 days, then transplantation). All surviving below-the-knee limbs demonstrated normal skin, fat muscle, and bone histology. «Safe cooling». Long-term survival of below-the-knee limbs cryopreserved by vitrification >Tg was significantly superior to that of limbs cryopreserved by DF or cooled to temperatures below the vitrification solution Tg. Below-the-knee limb was viable and able to support the rat’s weight, also demonstrating the regrowth of both total neuronal fibers and motor neurons.	[149]

Abbreviations: CPA—cryoprotectant agent; SF—slow freezing; USF—slow freezing with uncontrolled temperature; CSF—slow freezing with controlled temperature; DF—directional freezing; V—vitrification; Nano—nanowarming; PrOH—propylene glycol (1,2-propanediol); SUC—sucrose; ALB—albumin; EG—ethylene glycol; DMSO—dimethyl sulfoxide; SSS—synthetic serum substitute; LN—liquid nitrogen; FBS—fetal bovine serum; HSA—human serum albumin; SSCs—spermatogonial stem cells; OCT—optimal cutting temperature compound; O_2_—oxygen; CO_2_—carbon dioxide; VS55—EC/PrOH/DMSO/FA; VS49—49% vitrification solution; DP6—DMSO/PrOH; DIS—disaccharide; TRE—trehalose; AAHV—aortic allograft heart valve; PAHV—pulmonary allograft heart valve; HA—human albumin; G3PDH—glycerol-3-phosphate dehydrogenase assay; SVF—stromal vascular fraction; GLY—glycerol; EC—Euro-Collins; FA—formamide; X-1000—SuperCool X-1000; Z-1000—SuperCool Z-1000; mNPs—iron oxide nanoparticles with various coating, often silica-coated iron oxide nanoparticles (sIONPs); CCR—critical cooling rate; CWR—critical warming rate; N-MET—N-methylformamide; MP—3-methoxy-1,2-propanediol; POLY—polyvinylpyrrolidone; ALT—alanine aminotransferase; LDH—lactate dehydrogenase; UW—University of Wisconsin solution; VS83—VS55 in different concentration of components; VS70—VS55 in different concentration of components; TID—total indentation distance; IDI—indentation distance increase; AvgCID—average creep indentation distance; AvgED—average energy dissipated; PTH1—parathyroid hormone 1; OPG—osteoprotegerin; GAGs—glycosaminoglycans; DNA—deoxyribonucleic acid.

**Table 2:** Main differences between slow freezing and vitrification methods.

Parameter	Slow Freezing	Vitrification
working time [171]	more than 3 h	fast, less than 10 min
cost [171]	expensive, freezing machine needed	inexpensive, no special machine needed
sample volume (µL) [171]	100–250, wide range of sample volumes [6]	1–2
concentration of CPA [171]	low, more homogeneous distribution of CPAs, which need to diffuse through and permeate the extracellular space to take full effect [6]	high
risk of cryoinjury including ice crystal formation [171]	high due to the formation of extracellular ice [171]	low
post-thaw viability [171]	high	high
risk of toxicity due to CPA [171]	low	high
status of system [171]	closed system only	open or closed system
potential contamination with pathogenic agents [8,171]	low	high
manipulation skill [171]	easy	difficult
costly components [6]	equipment: variable-speed freezer [171]	operation costs
controllability/reproducibility [6]	high	low
main risks [6]	extracellular ice formation, osmotic damage	intracellular ice formation, CPA toxicity, fracturing, osmotic stress [6] risk of contamination with pathogenic agents [171]

**Table 3:** Main impact of cryoprotectants in sperm cryopreservation.

Cryoprotectant	Advantages	Disadvantages
Glycerol [178,188,189]	The most widely used penetrating cryoprotectant, preserves sperm viability and motility at relatively low concentrations	Toxic at high concentrations [190]
DMSO [181,184]	Offers better protection of motility compared to glycerol [13]	Toxic at high concentrations [190]
Trehalose [191,192]	Effective in maintaining high sperm motility and mitochondrial activity, allows reducing the concentration of penetrating protectants and increases sperm survival [193]	No data on its effect on sperm preservation for some animal species [193]
Cyclodextrin–cholesterol [178,194]	Maintains membrane stability during cryopreservation, allows reducing the concentration of penetrating cryoprotectants	Only used in combination with penetrating cryoprotectants
Egg yolk [178,189,194,195]	Maintains membrane stability during cryopreservation	Only used in combination with penetrating cryoprotectants, undefined composition

## Data Availability

The data that support the findings of this study are available from the corresponding author upon reasonable request, due to privacy.

## Supplementary Materials

The following supporting information can be downloaded at: https://www.mdpi.com/article/10.3390/ijms252011124/s1.
