# Supplementary material for: Current State and Challenges of Tissue and Organ Cryopreservation in Biobanking
Source: Int J Mol Sci. 2024 Oct 16;25(20):11124. doi: 10.3390/ijms252011124 (PMC11508709; doi:10.3390/ijms252011124)
Supplement: Supplementary file 1 [file ijms-25-11124-s001.zip › ijms-3186618-supplementary.pdf]

Supplementary Information

Current state and challenges of tissue and organ cryopreservation in biobanking

Irina V. Khaydukova [1], Valeria M. Ivannikova [1], Dmitry A. Zhidkov [1], , Nikita V. Belikov [1], Maria A. Peshkova [2], Petr S. Timashev [2], Dmitry I. Tsiganov [1,3] and Aleksandr V. Pushkarev [1,3,*]

[1] Bauman Moscow State Technical University, 105005, Russia; pushkarev@bmstu.ru (A.V. P.)
[2] Sechenov First Moscow State Medical University (Sechenov University), 119048, Russia
[3] Russian Medical Academy of Continuous Professional Education, 125993, Russia
* Correspondence: pushkarev@bmstu.ru

The methods are structured as follows:
1) The colour coding reflects the step: equilibration ➢freezing ➢storage ➢thawing ➢washing
2) Each step is represented by the **temperature,°C/ time**, min. If any of the parameters is unknown – it is replaced by "-" symbol
3) The abbreviations represent some common procedures: "h" – holding; "x3" – repeated 3 times; ↑ - stepwise increase of CPA; ↓ - stepwise decrease of CPA; - perfusion.

Therefore an example of the method represented as follows " **↑ice/90➢-/>8h ➢h10➢seeding➢−150/- ➢−80/7-30 days ➢ RT/30 (#1) or 37/5 (#2) ➢↓4/30 (x3)**" means "equilibration in CPA over ice for 90 minutes with perfusion in increasing steps of CPA concentration; freezing at unknown temperature for more than 8 hours; holding for 10 minutes; seeding; freezing to -150 °C for unknown time; storage for 7-30 days at -80 °C; thawing at room temperature for 30 minutes (1st protocol) or thawing at 37 °C for 5 min (2nd protocol); washing at 4 °C for 30 minutes three times in decreasing steps of CPA concentration".

References are the same as in the main article

**Tissue / Cryopreservation method**	**Object**	**CPA**	**Cryopreservation Protocol (temperature,°C/ time, min)**	**Reference**
**Ovarian Tissue**				
SF,	Human	PrOH/ SUC/ALB	**-/90➢−7/12➢h-➢seeding➢−150/-➢-90/-➢RT/30➢37/-**	[161]
V	Human	EG/DMSO/ SSS+SUC	**-/25➢-/15➢LN➢37/1➢RT/5➢HM**	[23], [24]
Mostly SF,	Human	-	**different methods**	[162]
Mostly SF,	Human	-	**different methods**	[163]
DF	Sheep	FBS/DMSO	**-/5➢−70/5h➢LN**	[30]
Not specified	Human	-	**different methods**	[164]
**Testicular Tissue**				
CSF,	Mouse	DMSO/SUC	**-/10➢–80/>8h➢LN➢37➢-/-**	[165]
USF	Human	DMSO/SUC/HSA	**-/15➢−80/>8h➢LN➢37/2➢37/-(x2)**	[166]
USF	Mice	DMSO/SUC	**RT/10➢−80/24h➢LN➢37/2➢37/-(x2)**	[35]
CSF	Human	DMSO/SUC/HSA	**ice/30➢0/- ➢h5➢−8/16➢seeding➢−40/64➢h10➢−70/5➢LN➢37/2➢-/5(x2)**	[166]
V	Human	DMSO/EG/HSA	**RT/10➢LN➢37➢h2➢37/5(x2)**	[166]

Solid-surface V	Mice	DMSO/EG	-/10➢-/5➢-/-➢LN➢37/2➢h2	[35]
**Heart**				
CSF AND USF	Wistar Rats	DMSO, O_2, CO_2	↑37/-➢1 above freezing point/-➢-/20➢ 37/-	[38]
USF	Minipigs	skinning solution	-/30➢4/>8h➢LN/2➢−80/1month➢-/-➢-/10(x3)	[41]
**Heart Valve**				
CSF	Human	DMSO	4/30➢−40/44➢−100/12➢LN/5years➢RT/5➢+37–40/5(x2)➢-/-	[42], [167]
CSF and V	Pig	VS55, VS49, or DP6/DIS/SUC/TRE	↑-/15,30,60➢−100/-➢−135/-➢>1day➢−100/5➢−25/3.40 or nanowarming −25/1.40 or −20/-➢↓-/10(x>2)	[168]
CSF	Human	DMSO	-/-➢LN➢RT/≥15➢37/≥15	[49]
Not specified	Human	-	-	[48]
**Vascular Tissue**				
CSF	Human	DMSO	−20/48➢−70/37➢LN➢<12months➢40/-➢-/-	[52]
Not specified	Human	DMSO	-/-➢LN➢↓37/-	[60]
CSF	Human	HA/DMSO	-/-➢−160➢VN/<5years➢0/-	[60]
CSF	Human	DMSO/ HA	−4/-➢−6/1➢−40/34➢−140/20➢VN/<5years➢RT/5➢37/5	[56], [169]
**Pancreatic Islet**				
V	Mouse, Pig, Human	EG/DMSO	21/10➢↑4/10(x2)➢LN/9month➢↓4/10(x2)➢+21/5➢-/15	[67]
CSF	Mouse, Pig, Human	DMSO	21/25➢−7.5/-➢seeding/15➢−40/0.25°C/min➢LN➢37/-➢0/30	[67]
Not specified	Human	islet cryopreservation solution	VN➢>3months➢-/-or 22/45➢37/45	[68]
CSF	Human, Mice, Rat	DMSO	↑22/5➢0/25➢−7.5/5➢seeding➢−40/0.25°C/min➢LN/4weeks ➢37/-➢22/-	[69]
V	Rat	EG/DMSO/FBS+SUC	RT/3➢RT/70sec(#1) or 40sec(#2)➢LN/>1week➢38.5/1(#1) or RT(#2)➢↓-/3-5(#1)	[70]
**Adipose tissue**				
CSF	Human	DMSO/TRE or TRE	4 or RT/10➢−30/2°C/min➢h10➢LN➢RT/2➢37/-	[77]
CSF	Human	not precised	-/15➢−80/1°C/min➢LN➢+37/3➢-/-	[72]
USF	Human		−20/-➢+37/6-10	[78]
USF	Human		−18/-➢slow thawing	[73]
CSF	Human	DMSO/HA	VN/1day-36 months	[170]
**Amniotic Membrane**				
USF	Human	GLY	−80/0.5-6 months➢RT/1h	[85]

USF	Human	GLY/RPMI or GLY/DMEM	−80/-➢RT/-➢-/-(x2)	[86], [171]
CSF	Human	DMSO/FBS	+4/10➢−80/76➢LN➢+37/-➢-/5	[172]
CSF	Human	GLY or DMSO	-/-➢−80 or <−140/-➢ LN/VN/-	[173]
**Cornea**				
CSF	Human	HSA/DMSO (#1); DMSO (#2); VS55 (#3); EC/ PrG /DMSO (#4).	↑-/3(x2) ➢4/10➢−120/-➢RT/1➢+37/-➢-/5(x3)(#1)or ↓-/5(#2,3,4)	[91]
Not specified	Human	Eusol-C	−78/-➢15 days - 12months	[92]
**Kidney**				
V	Rat	EC<br>DMSO/FA/EG/X-1000/Z-1000	0–4/20➢↑-/80➢h5➢↑➢h25➢-/-	[103]
V, Nano	Rat	EC + VS55+mNPs	0–4/-➢↑/15(x5)➢mNPs-/4-6➢0–4/-➢0/-➢–121/3➢h25➢−150/3➢h30➢nanowarming/-➢ ↓ice/15(x3)➢ ice/15-30	[100]
V, Nano	Rat	VS55	↑RT/15(x4)➢mNPs RT/10;-/-➢LN➢nanowarming/-➢↓-/~15(x3)	[101]
V,	Rabbit (Embryonic Kidneys)	EG/DMSO/N-MET/MP/POLY/X-1000 or Z-1000/ FBS	↑-/3(x2)➢RT/1➢LN➢3months➢↓37/1(x4)➢RT/5(x2)	[104]
V,	Microminiature Pig (Embryonic Kidneys)	FBS/EG/DMSO	↑ice/15(x2)➢LN/>3days➢42/1➢RT/3➢RT/5(x2)	[105]
**Liver**				
V, Nano	Rat	EG/sucrose/EC + mNPs	0–4/-➢−150/-➢1h-several days➢nanowarming➢ ↓	[111]
DF	Rat, Pig	UW/EG	4/3(rat) or 4/5min(pig)➢−40/-(rat) or 0/20➢−10/20➢-20/10➢−40/100(pig)➢-80/3-21days or LN/1-3days(rat)➢RT/5.5(rat)➢38/2.5(rat) or 38/17(pig)	[114]
CSF	Murine	EDTA+ UW/EG	/-5➢ -/ 3 ➢ −40/-➢−80/3-21 days (#1) or −LN/ 1-3 days (#2)➢RT/5.5➢+38/2.5	[114]
CSF	Pig	UW/EG	4/5 ➢-/15 ➢0/20➢−10/20➢ −20/10➢−40/100➢+38/17	[114]
**Cartilage**				
V	Pig	VS83,VS70 or VS55	↑ice/20 (x6)➢mNPs 4/-➢ −135/<br>≥ 1 week➢+37/- or nanowarming➢ ↓0/20 (x7)	[118]
CSF and V	Pig	DMSO (CSF); EG/DMSO/CS/ PrOH/SUC(V)	(#1): RT/30➢−80/105 ➢−80/21 days➢37/10 ➢4/30<br>(#2):-/- ➢ 1) 0/10; 2) 0/90; 3) –5/170 4) –10/160; 5) –196(LN)/21 days ➢+37/0.5➢↓4/30 (x2)	[117]
USF and V	Pig	-(USF)	Freezing: (LN /15 ➢+37/-)(x4)	[116]

		DMSO/EG/PrOH(V)	Vitrification: ↑ 0/90 (#1) or −5/170 (#2) or −10/160 (#3) ➢LN/>8h➢37/-➢4/30	
**Bone**				
USF	Mini-Pig	-	**−80/1 month**	[174]
USF	Mini-Pig	-	**−80/≥ 1 month**	[175]
USF	Human	-	**−80/≥ 1 year**	[124]
USF	Human	DMSO	**-/-➢ −80 or freeze-dried ➢-/-➢-/-**	[126]
USF	Human		**Storage −23 (#1) or −80 (#2)/2-17 months➢RT/2h**	[176]
**Trachea**				
USF	Pig	DMSO	**-/-➢ISO antifreeze box➢−80/-**	[136]
CSF	Pig	DMSO, HSA	**-/-➢ −160 VN/13 months**	[177]
CSF	Rabbit	DMSO	**-/-➢−40/1°C/min➢−100/12➢ VN −170/ 7-95 days➢RT/5-7 ➢37–40/8-10➢↓4/-(x4)**	[133]
CSF	Rat	FBS/ DMSO/ SUC	**-/-➢4/60➢−80/76 ➢−196/1 month ➢ 37/- ➢-/-**	[134]
**Complex**				
DF	Rat	FBS/DMSO/sucrose	**4/15➢−10/7➢h14➢ −40/110➢−140/10 ➢LN➢-/8➢-/15 min**	[142]
DF	Rat	DMSO/FBS/TRE	**-/-➢−70/0.5°C/min➢−80/7–30 days➢+65/2-3➢ +38/- ➢** 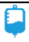 **-/5**	[143]
V	Rat	DMSO/EG/TRE	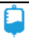 **↑-/2 ➢−208/-➢LN −80/7-30 days➢RT/10➢38/-➢** 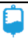 **-/5**	[143]
**Skin**				
CSF	Human	DMSO	**4…8/-➢−60/56➢−120/12➢−150/6➢VN➢4…8**	[148]
Not specified	Human	DMSO	**-/-➢−80**	[147]
Not specified	Human	DMSO	**➢LN/5 years➢ice/5 ➢RT/-➢-/-(x3)**	[149]
Not specified	Human	-	**Protocol is not mentioned**	[152]
**Brain and nerve**				
USF	Human	DMSO/GLY	-	[154]
USF	Rat	DMSO/GLY	+2/- ➢-16/2 days➢on ice➢-80/-➢7 weeks➢➢➢➢➢➢➢➢➢➢➢	[154]
CSF	Mice	FBS/DMSO	−80/4h➢LN➢>1 week-3 months➢37/-	[178]
USF	Human	methylcellulose, EG, DMSO, Y27632	RT/0.16-5h➢-80/24h➢LN➢37/-	[156]
CSF	Pig	-	-80/-➢7–8 days➢37/1	[157]
**Muscle or groin flap**				
CSF	Rat	DMSO/GLY/glucose	-4.2°C/-➢seeding➢-40/-➢-70/-➢LN➢1-2 weeks➢10/5	[159]
V AND CSF	Rat	DMSO/ EG/ Trehalose/ HSA(V)	A list of cryopreservation methods is given in the paper	[158]
